# AI-enabled workforce integration: blended human resource contribution rate in Chinese companies

**DOI:** 10.3389/frai.2025.1645172

**Published:** 2025-07-09

**Authors:** Kexin Zhang, Cisheng Wu, Manman Ge, Teng Liu

**Affiliations:** School of Management, Hefei University of Technology, Hefei, Anhui, China

**Keywords:** human resources, formal employees, flexible workers, intelligent machine workers, contribution rate, backward (BP) neural network, mean impact value (MIV) algorithm, nonlinear relationships

## Abstract

**Introduction:**

With the development of AI technology, the employment mode of companies is undergoing unprecedented changes.

**Methods:**

The paper defines the composition of blended human resources of a company as three types of formal employees, flexible workers and intelligent machine workers, constructs a blended human resource contribution rate calculation method based on BP-MIV, and analyzes the data of automobile manufacturing companies in 2022.

**Results:**

The results show that the contribution rate of blended human resources to company performance is 73.81%. Among them, the contribution rate of formal employees is 19.55%, while flexible workers and intelligent machine workers, despite their significantly smaller proportion in number compared to formal employees, have contribution rates of 20.26% and 34.00%, respectively. In further discussions, the calculation results of the blended human resource contribution rate based on the production function method were compared with those based on the BP-MIV method.

**Discussion:**

The findings indicate that the BP-MIV-based calculation method exhibits certain advantages in capturing nonlinear relationships, such as the synergistic effects of various types of blended human resources on company performance. This study attempts to propose a preliminary theoretical framework and methodological approach for blended human resource management research in the AI era.

## 1 Introduction

With the continuous innovation of digital and intelligent technology, the company’s employment mode is undergoing unprecedented changes. Based on digital technology, flexible workers have evolved into new forms such as shared workers, platform workers, and expert think tanks (Guo, 2022; Wood et al., 2019), which have become new choices for companies (Tu, 2018). *Harvard Business Review* suggests that integrating and managing this emerging blended workforce will be a major challenge for human resource management in the future (Gratton, 2024). In addition, AI technology drives machines to become human-like agents (He and Zhu, 2023), competent in human work (Zhao et al., 2020), and teammates rather than tools (Seeber et al., 2020). New forms such as intelligent machine coworkers, human-machine hybrid teams, and robot bosses emerge in companies (Liu et al., 2020; Couto et al., 2023). The above phenomena reveal that the concept of blended workforce in the digital economy needs to be further broadened into blended human resources of companies covering intelligent machine workers, flexible workers and formal employees. Some studies have shown that changes in the structure of human resources help companies reduce costs and enhance organizational flexibility and competitiveness, but they also bring challenges to organizations such as complexity of relationship management, process reengineering, reshaping the labor division system, and loss of human capital (He et al., 2021; Zhang et al., 2021; Wang X. et al., 2022). Therefore, the impact of blended human resources on company performance needs to be further explored. Calculating the contribution rate of blended human resources to company performance can illustrate the extent to which blended human resources influence company performance through objective data, and it can compare the influence of different types of employees on company performance, providing decision-making information for companies to optimize resource allocation. Therefore, developing a method to calculate the contribution rate of blended human resources to company performance has emerged as a new challenge for human resource management in the digital economy era.

The classical calculation method of the contribution rate of human resources is to construct a production function to represent the relationship between the inputs of human resources and material resources and the outputs such as performance of the company as well as the economic growth of the country, expressing the contribution rate of human resources through the parameters or coefficients in this function (Ma, 2021; Li et al., 2004). Wu et al. divided the input of human resource into two variables, labor force quantity and human capital level, and further investigated the contribution rate of human capital investment to company performance as well as national economic growth (Wu, 2018; Wu, 2012). Zhou and Hu (2019) constructed the “main factor analysis model” basing on Paul Romer’s new growth theory, and computed the contribution rate of several factors, including the number of labor force to the national economic growth. The advantages of the above method of calculating the contribution rate by constructing a production function model and applying mathematical principles are that (1) the model has a clear economic meaning, produces an accurate solution and has high stability; (2) the method is relatively mature in the application of the problem of evaluating the contribution rate of human resources. The disadvantages are that (1) this type of calculation method is subjectively constructed by the researcher, which is difficult to adapt to highly nonlinear and complex socio-economic systems; (2) the validity of the method is greatly affected when the statistical data are incomplete or erroneous, and the actual situation does not conform to the model assumptions (Song and Guo, 2017).

Thus, quantitative analysis methods of AI are applied to the problem of computing contribution rate, including neural network systems, fuzzy logic, approximate reasoning and genetic algorithms. This type of method corresponds to the human brain, has self-learning and self-adaptive capabilities, and is able to deal with complex nonlinear problems through uncertain and imprecise data to draw more robust conclusions (Song and Guo, 2017). Guo et al. (2008) applied fuzzy neural networks to analyze the contribution rate of education to economic growth, via calculating the contribution rate of education to human capital as well as the contribution rate of human capital to the national economic growth, respectively, and then multiplied the results. He et al. (2016) applied fuzzy neural networks to the calculation of the contribution rate of the production factors to the regional economy, and the production factors included human capital. The calculation method of the above two studies is to express the contribution rate of production factors to economic growth through the weights from input layer to hidden layer after constructing the fuzzy neural network model. Besides, the Mean Impact Value (MIV) algorithm is another method to compute the impact weights of neural network input neurons on output neurons, which is widely used in the problem of contribution rate calculation in various fields such as medicine, geology, management, etc. (He et al., 2013; Wang et al., 2024), and the MIV is considered to be the optimal index for evaluating the relevance of neural network input parameters (Yang et al., 2018; Sun et al., 2012).

Compared with the traditional human resource structure dominated by formal employees, the relationship between blended human resources and company performance is more complex. Although the research on the calculation of the contribution rate of traditional human resources to company performance has already possessed rich results, how to compute the contribution rate of the blended human resources to company performance has not yet attracted much attention, and there are many problems that need to be explored: for example, the relationship between blended human resources and company performance is probable to present nonlinearity, how to fit it effectively through the model? How to quantify the contribution of various types of blended human resources? Blended human resource involves a large amount of data at the company level, which may be difficult to collect accurately and completely, how to ensure the feasibility of the computing method and the validity of the results? Based on the above problems, the neural network algorithm has certain superiority. However, existing relevant studies tend to reflect the contribution of various production factors to economic growth through the weights from the input layer to the hidden layer of the neural network. Although this is a common practice to quantify the influence of input variables on output variables in neural network models, it should be noted that the independent variables are mapped from the input layer to the hidden layer and then to the output layer, in addition to the processing of weights and bias values, they will also be processed by the nonlinear function (activation function). The weights only determine the “strength” of the input signals during transmission, while the activation function determines how these signals are “transformed” and “activated,” which in turn affects the calculation and final output of the subsequent layers. In addition, each layer of the neural network performs further feature extraction and combination based on the output of the previous layer (as processed by the activation function). This means that as the depth of the network increases, the effects of the initial input variables are transmitted layer by layer, mixed and may be reflected in the final output in a highly nonlinear manner. The nonlinear nature introduced by the activation function, as well as the complex hierarchical structure and information transfer mechanism within the neural network, makes it a significant limitation to reflect the contribution of each factor of production to economic growth only through the proportion of weights.

Based on the above, this paper constructs a BP neural network and use the MIV algorithm to calculate the contribution rate of blended human resources to company performance. The marginal contributions of this paper are: first, it enriches the connotation of human resources by defining the structure of blended human resources; second, it is the combination of BP neural network and MIV algorithm, which is innovatively applied in the problem of computing the contribution rate of blended human resources; Third, the contribution rate of blended human resources is derived based on the data of China’s automotive industry in 2022, thus reflecting the actual efficiency and output level of various types of blended human resources under the current level of technological development in China.

## 2 Connotation of blended human resources and indexing features extraction

### 2.1 The formation and connotation of blended human resources

The research on company’s blended human resources can be traced back to The Shamrock Organization Theory proposed by Handy (1990). The “shamrock” refers to a company’s human resources consisting of formal employees, for example specialists, senior technicians and core managers, the contractual fringe, and the flexible labor force. However, with the development of digital and intelligent technology, people increasingly use online platforms to sell their labor (Wood et al., 2019), completing small tasks or “gigs” for consumers or businesses through platform-based matching (Gandini, 2019). This emerging gig economy led to a further diversification of company human resources. Economists from Harvard University and Princeton University predicted that the future workforce will be a mix of full-time employees, consultants, contractors, freelancers, part-time workers, and other temporary workers (Mulcahy, 2016). On this basis, Gratton (2024) proposed the concept of blended workforce, which argues that organizations are made up of full-time employees, highly skilled freelancers, temporary contract workers, and outsourced contract workers, and emphasizes that the most sought-after skills in many fields (especially technology, data science, and machine learning) are in the hands of freelancers.

On the other hand, the development and application of AI technology has enabled industrial robots to be automatically controlled, reprogrammable and versatile intelligent machine rather than a simple assembly-line robot arm that performs specific tasks following an edited program or relying on the built-in power and control mechanisms to accomplish certain actions (Zhang et al., 2023). Intelligent devices (such as robots) are able to act as human-like agents (Gao and Liu, 2018; He and Zhu, 2023), capable of performing human jobs (Zhao et al., 2020), and becoming widespread in various companies as a replacement for traditional labor (Guo, 2022). Dellermann et al. (2019) argue that while AI performs well in some well-defined tasks, such as playing chess or recognizing objects on an image, the development of general-purpose AI (AGI) capable of solving multiple tasks at once is still a long way off, and thus the most likely model for the division of labor between humans and machines in the coming decades is hybrid intelligence. Hybrid Intelligence (HI), as a division of labor paradigm between humans and machines, aims to build a synergistic symbiotic system between humans and AI in order to leverage the complementary strengths of both to achieve transcendence over the capabilities of a single entity (Mestre, 2024). Based on these phenomena, 65 collaborating scientists initiated an international initiative to explore the advantages and potential risks of machines as teammates (MaT). The study suggests that future research needs to design for human-machine collaboration, such as the division of labor between machine teammates and human teammates (Seeber et al., 2020). Adamik and Walecka (2024) proposed a conceptual model of the human-technology dichotomy, which argues that in the context of Industry 4.0, human resources in the socio-economic structure evolve to the human-technology contamination approach stage, where humans and AI work as collaborators. Governments in countries like South Korea are even attempting to impose a “robot tax.”

Therefore, some scholars have proposed the Four-Leaf Clover Organization Theory on the basis of The Shamrock Organization Theory by studying the new blended labor organization form. The so-called “four-leaf” consists of four parts: professional core workforce, flexible workforce, intelligent machine workforce, and producer and seller workforce (He et al., 2021), and He et al. (2022) applied the labor force ecosystem theory to interpret the organization’s diverse and blended labor force structure.

Although the existing literature has carried out more in-depth research on various types of labor and initially discussed the new blended labor forms, a consistent conclusion has not yet been reached, and a more in-depth exploration and a clear definition of the trend of blended human resources in the context of AI technology are still needed. Clarifying the structure of blended human resources can provide novel theoretical perspectives and unique solutions for revealing the contemporary problems of diversified labor division and work integration, and provide decision-making information for company resource allocation.

In view of this, this paper defines an organization’s blended human resource consists of three parts: formal employees, flexible workers, and intelligent machine workers. Among them, formal employees, typically comprising senior experts, technicians, and managers, bear important organizational functions and constitute the most stable core human resources within the company. Flexible workers refer to all personnel employed by the company that are distinct from standardized labor relationship contracts, specifically including part-time workers, labor dispatch employees, labor outsourcing employees, shared employees, short-term labor contract workers, expert consultants, platform workers, etc. Intelligent machine workers primarily consist of robots and other smart machine equipment.

### 2.2 Extraction of indexing features

Based on the theory of economic growth, the source of growth in company performance is the input of factors of production, including material and human resources, as well as technological progress. Therefore, the extraction of the indexing features affecting the performance of the company is carried out from the factors of production and factors influencing technological progress. Indexing feature of company performance use the operating revenue of the company.

According to the definition of blended human resource composition in 2.1, the indexing features of blended human resources in production factors are categorized into the number of formal employees, the number of flexible workers and the number of intelligent machine workers. In addition, the indexing feature of material resources is expressed using the stock of fixed assets (Wu, 2012; Guo et al., 2008). Based on the endogenous growth theory (Romer, 1990), technological progress originates from within the company: (1) technicians among formal employees, who are engaged in research and development, are committed to improving the technological level of the company; (2) among flexible workers, high-end laborers such as consultants and experts also contribute to the technological innovation of the company by providing technological consulting and so on; (3) Intelligent machine workers, on the one hand, replace humans in performing “codable” or “predictable” routine tasks (Yang et al., 2022), allowing R&D workers to devote more time to technological innovation; on the other hand, they generate a large amount of data in the course of their operations, which reflects multi-dimensional information such as business processes, market dynamics and user behavior, and promotes technological progress through the “multiplier effect” (Rogers and Mcclelland, 2014). In summary, the combination of various types of human and material resources (fixed assets) to promote the technological progress of the company. Therefore, the indexing features of the technical progress factor are the same as those of the production factor. Therefore, the results of extracting indexing features are shown in Table 1.

**Table 1:** Indexing features of the neural network model.

Factors	Meaning	Indexing features
Formal employees	Senior experts, technicians, managers, etc.	Number of formal employees
Flexible workers	Part-time workers, labor dispatch workers, labor outsourcing workers, shared workers, short-term labor contract workers, expert consultants, platform workers, etc.	Number of flexible workers
Intelligent machine workers	Robot-based intelligent machine equipment, etc.	Number of intelligent machine workers
Fixed assets	Amount invested in fixed assets of the company	Original value of fixed assets
Company performance	Economic size and profitability of the company	Operating revenue of the company

Source(s): created by authors.

## 3 BP-MIV computing method of contribution rate of the blended human resource

**Figure 1:**
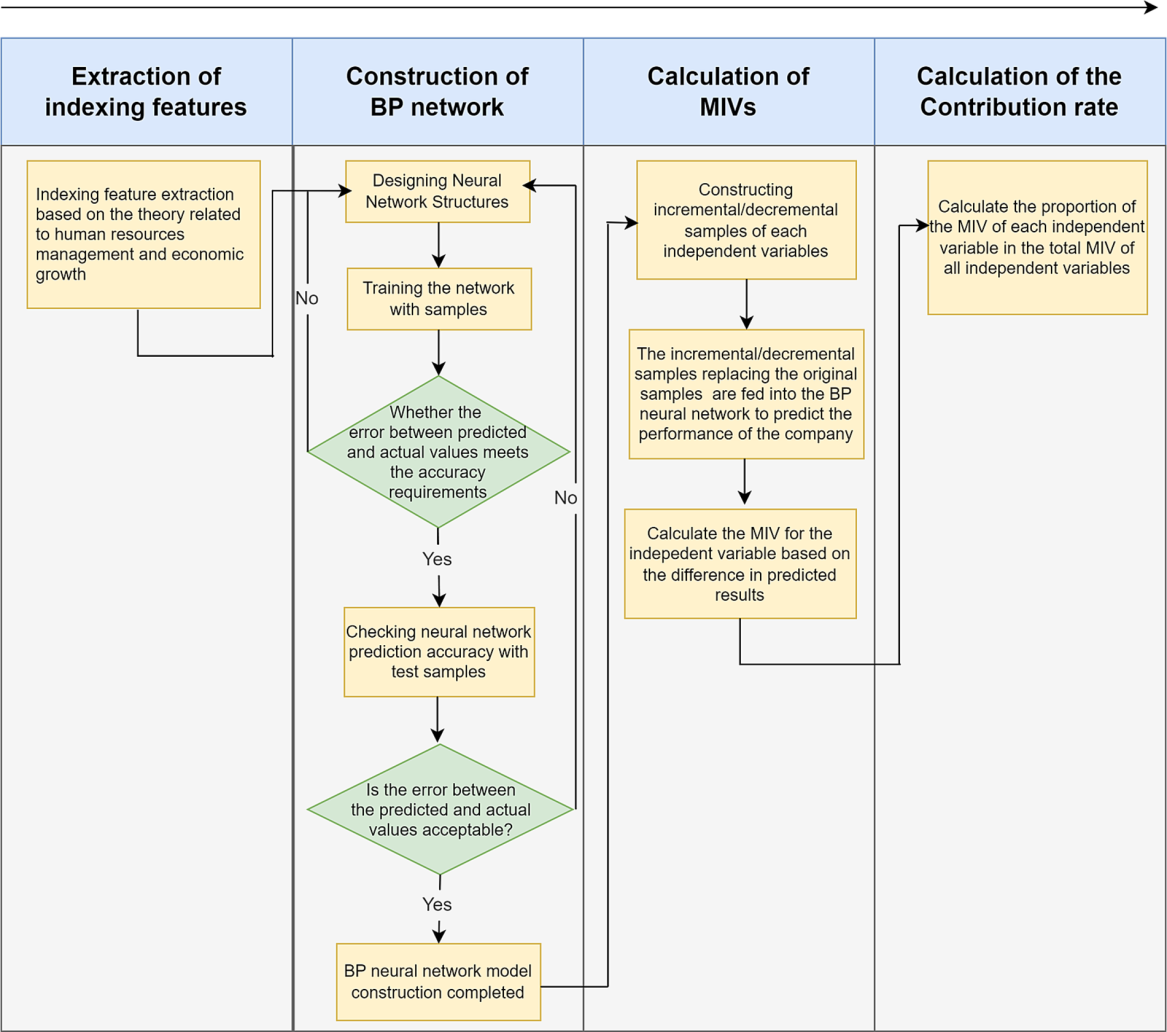
Computing method of company blended human resource contribution rate based on BP-MIV. Source(s): created by authors.

After completing the indexing feature extraction, the neural network is constructed to reflect the mapping relationship between various types of indexing features, and then the contribution rate is calculated using the MIV algorithm, the specific process is shown in Figure 1.

### 3.1 The construction of BP neural network model

Backpropagation neural network (BP neural network) is a multi-layer forward neural network based on the error backpropagation algorithm researched and designed by Rogers and Mcclelland (2014), which is one of the widely used neural networks at present. The BP neural network consists of one input layer, one or more hidden layers and one output layer. This structure establishes a complex connection network between neurons at various levels, which can learn and store a large number of input–output pattern mapping relationships. Therefore, this paper constructs a BP neural network prediction model for the relationship between production factors and company performance, with the following steps:

#### 3.1.1 Determine the input layer and output layer

Based on the results of indexing feature extraction, the input layer of this BP neural network is the number of various types of blended human resources (specifically the number of formal employees, the number of flexible workers, and the number of intelligent machine workers) and the value of fixed assets, which are the four independent variables, and the operating revenue as an alternative index of company performance is the dependent variable. Therefore, the number of neurons in the input layer is 4 and the number of neuron in the output layer is 1.

#### 3.1.2 Determine the hidden layer structure

The number of hidden layers should be chosen based on the complexity of the problem. A single hidden layer is generally sufficient for tasks that are linearly separable or exhibit simple nonlinear patterns. For more complex nonlinear relationships, a dual-hidden-layer structure can significantly enhance the model’s learning and modeling capabilities, providing stronger expressive power than a single hidden layer (Li et al., 2019; Hou et al., 2020; He et al., 2024). It is important to note that increasing the number of hidden layers does not necessarily lead to improved network performance. On the contrary, an overly complex multi-hidden-layer structure may introduce overfitting issues (Yang and Chen, 2013). Therefore, it is recommended to adopt a dual-hidden-layer architecture as the initial choice for addressing complex nonlinear problems, with the final network depth determined based on experimental validation. The number of neurons in the hidden layers is initially estimated using the empirical Equation 1 to determine a reasonable range (Hu and Zhou, 2023):


(1) $$N=\sqrt{n+m}+a,a=1\sim 10$$

**Figure 2:**
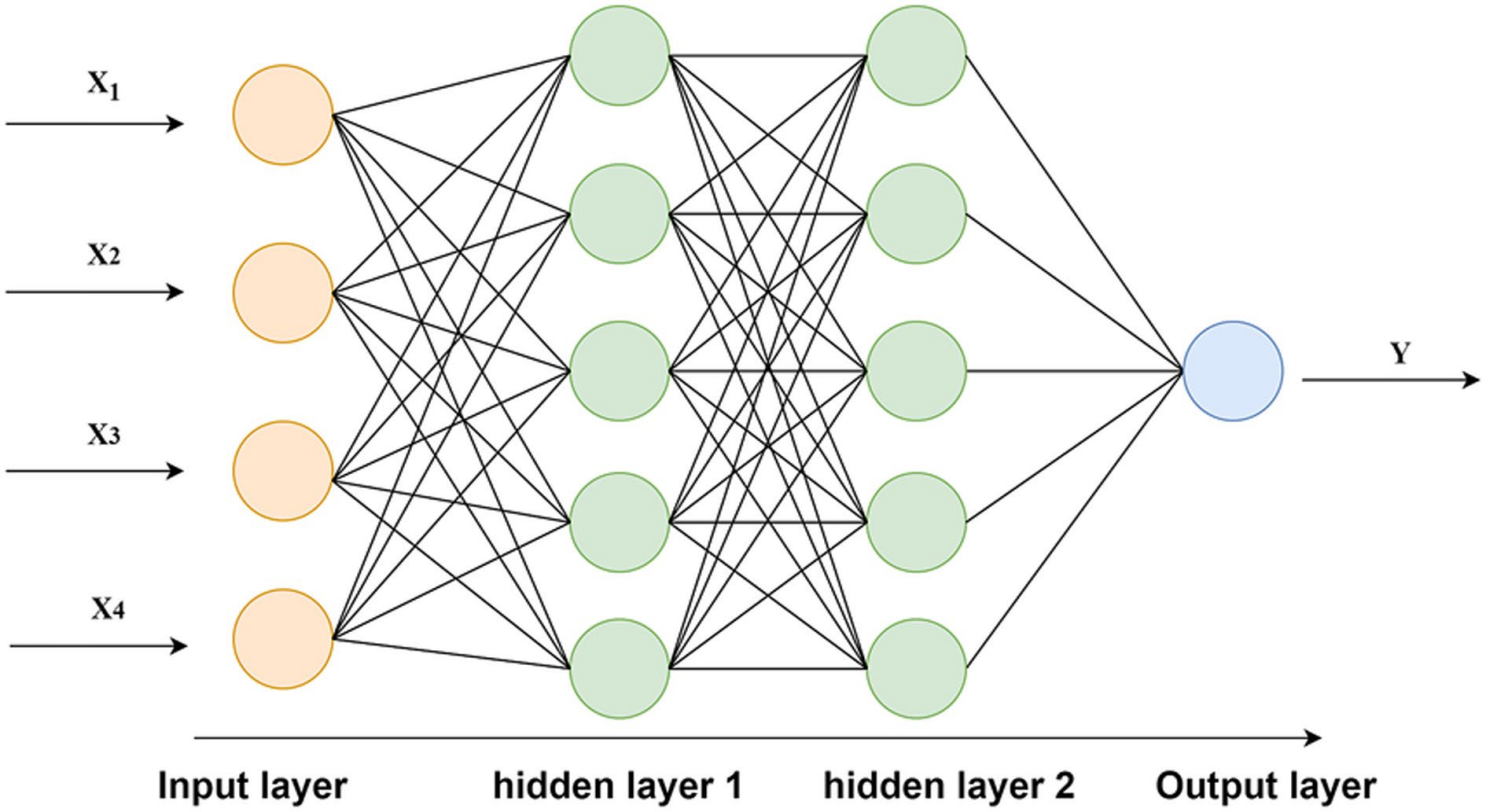
Structure of the BP neural network. Source(s): created by authors.

Where n represents the number of input-layer neurons and m denotes the number of output-layer neurons. The optimal number of hidden-layer neurons is then determined by comparing the model’s average prediction error across different parameter settings. After repeated experiments, it was found that the neural network achieved the smallest error when structured with 2 hidden layers, each containing 5 neurons. Consequently, the final architecture was set to 2 hidden layers with 5 neurons per layer, as illustrated in Figure 2. And the activation function of each hidden layer is the hyperbolic tangent S-shaped tansig function and the activation function of the output layer is pure linear purelin function.

### 3.2 Calculation of contribution rate based on MIV

MIV is the change value caused by the effect on the dependent variable after varying the independent variable, as an index to determine the size of the impact of the input neurons on the output neurons, with the sign representing the direction of the impact (positive or negative) and the size of the absolute value representing the relative importance of the impact. The implementation process of the MIV algorithm is as follows:

1. Construct the input matrix X of the test sample, where the m -th row represents the value of the m -th sample, and the i -th column represents the value of the i -th independent variable in all samples; referring to the classical calculation of MIV (He et al., 2013; Wang et al., 2015; Xie et al., 2019; Hu and Zhou, 2023), add or subtract 10% to the original value of the i -th independent variable in the matrix X , respectively, and keep the value of the other independent variables unchanged, constituting the incremental sample $X_{i}^{+}$ and the decremental sample $X_{i}^{-}$ , as shown in Equations 2, 3;

(2) $$X_{i}^{+}=[\begin{array}{c} \begin{array}{c} x_{11}\\ x_{21} \end{array}\;\begin{array}{cc} \cdots & x_{1i}(1+10\%)\\ \cdots & x_{2i}(1+10\%) \end{array}\;\begin{array}{cc} \cdots & x_{14}\\ \cdots & x_{24} \end{array}\\ \;\begin{array}{c} \vdots \\ x_{m1} \end{array}\;\begin{array}{cc} \vdots & \vdots \\ \cdots & x_{\mathrm{mi}}(1+10\%) \end{array}\;\begin{array}{cc} \vdots & \vdots \\ \cdots & x_{m4} \end{array} \end{array}]$$

(3) $$X_{i}^{-}=[\begin{array}{c} \begin{array}{c} x_{11}\\ x_{21} \end{array}\;\begin{array}{cc} \cdots & x_{1i}(1-10\%)\\ \cdots & x_{2i}(1-10\%) \end{array}\;\begin{array}{cc} \cdots & x_{14}\\ \cdots & x_{24} \end{array}\\ \;\begin{array}{c} \vdots \\ x_{m1} \end{array}\;\begin{array}{cc} \vdots & \vdots \\ \cdots & x_{\mathrm{mi}}(1-10\%) \end{array}\;\begin{array}{cc} \vdots & \vdots \\ \cdots & x_{m4} \end{array} \end{array}]$$

2. The incremental samples $X_{i}^{+}$ and the decremental samples $X_{i}^{-}$ are inputted into the BP neural network to obtain 2 sets of predicted output vectors $Y_{i}^{+}$ and $Y_{i}^{-}$ , as depicted in Equations 4, 5;

(4) $$Y_{i}^{+}={\left[y_{i1}^{+}\;\;y_{i2}^{+}\cdots y_{\mathrm{im}}^{+}\right]}^{T}$$

(5) $$Y_{i}^{-}={\left[y_{i1}^{-}\;y_{i2}^{-}\;\cdots y_{\mathrm{im}}^{-}\right]}^{T}$$

3. The average difference between $Y_{i}^{+}$ and $Y_{i}^{-}$ is the weight of the i -th production factor;

${\mathrm{MIV}}_{i}=\frac{1}{m}\sum_{j=1}^{m}(y_{\mathrm{ij}}^{+}-y_{\mathrm{ij}}^{-}).$

4. The contribution rate of the independent variable $C_{i}$ is obtained by dividing the absolute value of ${\mathrm{MIV}}_{i}$ of the i -th factor by the sum of the absolute values of ${\mathrm{MIV}}_{i}$ of all factors (see Equation 6).

(6) $$C_{i}=\frac{|{\mathrm{MIV}}_{i}|}{\sum_{i=1}^{n}|{\mathrm{MIV}}_{i}|}$$

## 4 Empirical analysis

### 4.1 Data sources

The number of intelligent machines and flexible workers in the automobile manufacturing industry is at a relatively advanced level (Wang X. et al., 2022), so the data of each company in the automobile manufacturing industry in 2022 is selected as the experimental sample. A total of 72 company samples are obtained by removing the companies with missing data. The data of the employee number, fixed assets, and revenue of each company can be obtained directly from the reports of China Stock Market andAccounting Research (CSMAR) Database.^1^ The rest of the data were estimated through relevant academic literature and secondary data. Based on the statistical results of China Flexible Worker Development Report (2022) (Li and Li, 2022), combined with the relevant conclusions of the flexible labor research (Mulcahy, 2016), the percentage of flexible workers in manufacturing companies in 2021 is 14.73%, with an annual growth rate of 18.79%, due to which it is estimated that the percentage of flexible workers employees in 2022 in each company is about 17.50%. In addition, the intelligent machine workers in the automobile manufacturing industry are mainly industrial robots. Based on the official data of International Federation of Robotics (IFR) (Wang X. et al., 2022), the industrial robot penetration degree at the company level is calculated (Wang J. et al., 2022; Acemoglu and Restrepo, 2018; Wang and Dong, 2020), and then the number of industrial robots in each company is calculated as the value taken for the number of intelligent machine workers (the specific process is shown in Appendix A).

**Figure 3:**
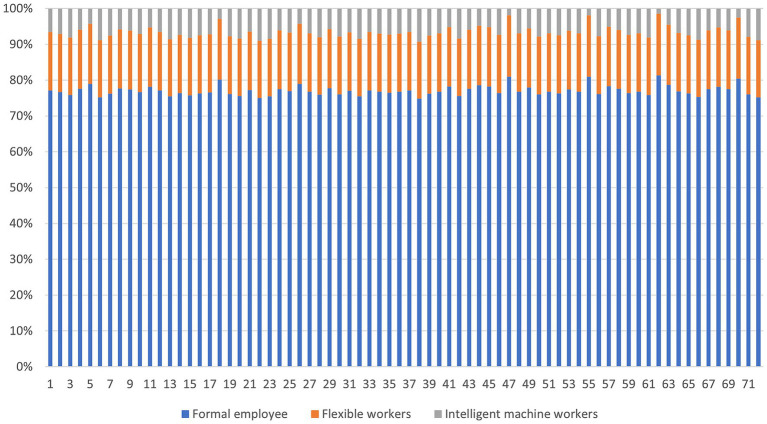
Proportion of various types of blended human resources in automotive manufacturing companies in 2022. Source(s): created by authors.

According to the calculation results, as shown in Figure 3, the proportion of formal employees in the blended human resource of automobile manufacturing companies is about 70–80%, followed by flexible workers accounting for about 10–20%, and finally intelligent machine workers accounting for <10%. Therefore, it can be inferred that during this period, Chinese automotive manufacturing companies were still in the initial stage of a blended human resources model dominated by formal employees.

### 4.2 Computing process

**Figure 4:**
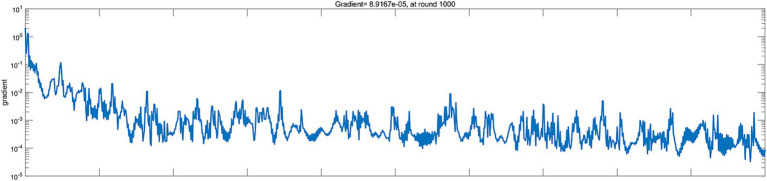
Training Status of the BP neural network. Source(s): created by authors.

Firstly, a BP neural network for predicting corporate performance was constructed based on the aforementioned steps, with 52 sets of samples from 72 companies in China’s automobile manufacturing industry being used for neural network training. The training status is shown in Figure 4, indicating that the network quickly learned and adjusted its parameters. As the number of iterations increased, the rate of error decrease slowed down and eventually stabilized, reflecting the process of the network gradually approaching the optimal solution. The final error value remained at a low level, indicating that the training effect was favorable, and the error was well controlled. Furthermore, there were no obvious signs of overfitting or under fitting in the figure, further proving the stability and effectiveness of the training. Based on this figure, the training status of the BP neural network is satisfactory, with high training efficiency and accuracy.

**Figure 5:**
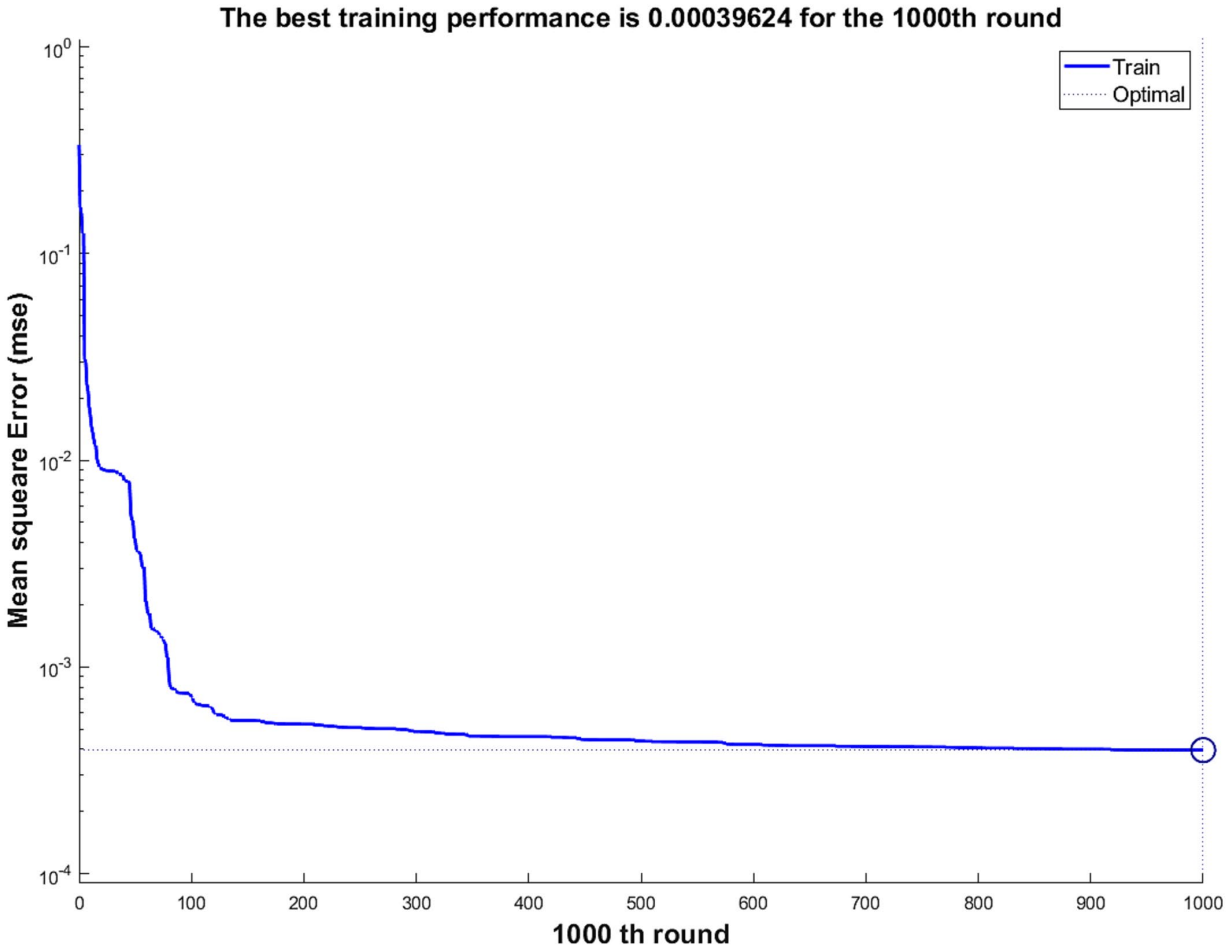
Training performance of the BP neural network. Source(s): created by authors.

The training performance is shown in Figure 5. After 1,000 training iterations, the mean squared error of the neural network is 0.00039624, indicating that the average squared deviation of the network’s predictions is very small, and the training results are acceptable.

**Figure 6:**
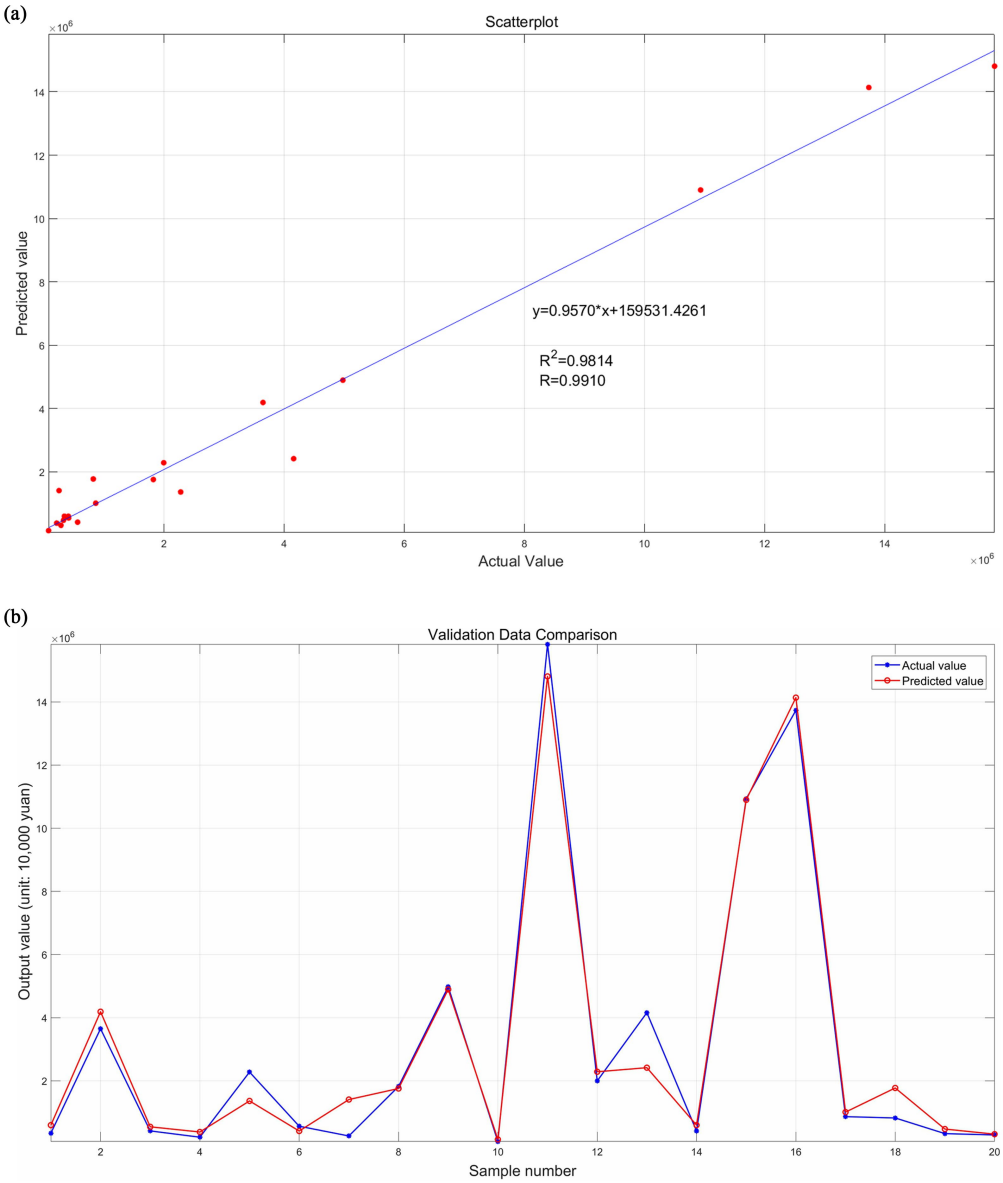
Comparison of predicted and actual values of test samples. **(a)** Presents the scatter fit plot; **(b)** presents the validation data comparison plot. Source(s): created by authors.

**Figure 7:**
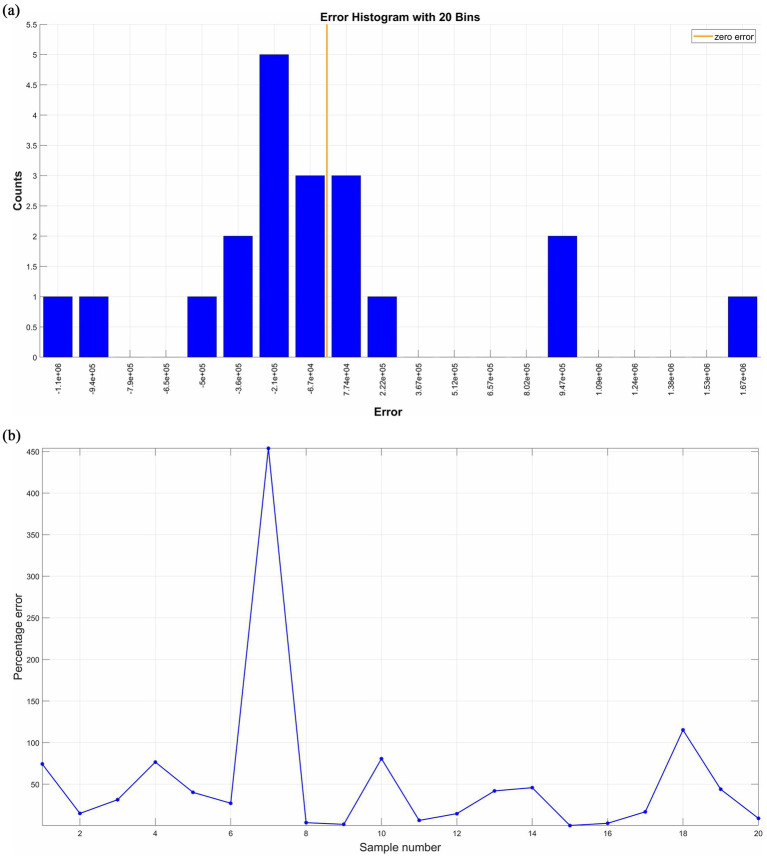
Errors between predicted and actual values of test samples. **(a)** Presents the error histogram with 20 bins; **(b)** presents the distribution of the percentage of error. Source(s): created by authors.

Secondly, the remaining 20 groups of the 72 samples are used as test samples and input into the trained neural network to predict company performance. The prediction accuracy of this neural network is reflected by comparing the predicted values with the actual values, and the results are shown in Figures 6, 7. The scatter fit plot in Figure 6 shows that most of the data points of the predicted values are closely distributed along the fitted line, which visualizes the close relationship between the predicted and actual values. Only a few points deviate far from the line, indicating that the prediction errors of these samples are relatively large, but overall, the R^2^ is 0.9814, indicating that the model can explain 98.14% of the data variability, with excellent fitting and high prediction accuracy. The validation data comparison plot in Figure 6 further illustrates that the predicted (red line) and actual (blue line) values are more consistent in the overall trend, with significant error fluctuations at samples 5, 7, 13, and 18. By grouping the error data into 20 equal-width intervals (i.e., bins) in Figure 7, it can be seen that the error is mainly concentrated around zero (i.e., the height of the center bin is significantly higher than that of the other bins), and according to the formula Percentage of Error = |(Actual Value - Predicted Value)/Actual Value*100%|, the overall percentage of error is low and the distribution is relatively evenly spread out, indicating that the error is within the acceptable range.

According to the steps in 3.2, the MIV algorithm is used to calculate the contribution rate of the four input variables (1-formal employees, 2-flexible workers, 3-intelligent machine workers, and 4-fixed assets), and the results are shown in Table 2 (two decimal places are reserved). Based on the data of automobile manufacturing companies in 2022, the contribution rate of fixed assets to company performance in this industry is 26.19%, and the contribution rate of blended human resources to company performance is as high as 73.81%, of which, the contribution rate of formal employees is 19.55%, the contribution rate of flexible workers is 20.26%, and the contribution rate of intelligent machine workers is 34.00%.

**Table 2:** Contribution rates of various production factors (calculated by BP-MIV method).

Label	1	2	3	4
Input variable	Formal employees	Flexible workers	Intelligent machine workers	Fixed assets
Contribution rate	19.55%	20.26%	34.00%	26.19%

Source(s): Created by authors.

### 4.3 Results and discussion

Combining the analysis with the number share (Figure 3) and contribution rate (Table 2) of each type of blended human resources in automobile manufacturing companies, the following conclusions can be drawn:

The contribution of blended human resources to company performance far exceeds that of fixed assets. This indicates that the optimized allocation and efficient utilization of blended human resources is the key to company performance improvement in the automobile manufacturing industry. Companies should continue to optimize the structure of blended human resources and explore the appropriateness of the weighting ratios of formal employees, flexible workers and intelligent machine workers in order to maximize the utilization and output of employees.

Although formal employees account for the highest number of employees in each company, generally about 70–80%, and are the stable human resource foundation of the company, their contribution rate is the lowest compared to the other two types of workers. The reasons for the relatively low contribution rate of formal employees are manifold, including the solidification of roles and responsibilities, the limitations of incentive mechanisms, the singularity of career development paths, the low adaptability to change, the high cost of teamwork and communication, and the slow pace of updating skills and knowledge. Although the contribution rate of formal employees is relatively low, they have an irreplaceable role in the company’s cultural heritage, team stability and long-term project execution (He et al., 2021). Therefore, companies need to re-examine the value of formal employees and stimulate their potential and creativity through role transformation and value reshaping, for example, by providing diversified career development paths, establishing a fair incentive mechanism, and encouraging a culture of innovation and learning, so as to help formal employees adapt to the requirements of the digital and intelligent era and create greater value for the company.

The number of flexible workers only accounts for about 10–20%, but their contribution rate to the company performance ranks second, slightly higher than that of formal employees, which reflects the higher efficiency and output capacity of flexible workers than that of formal employees in the digital and intelligent era. It is noteworthy that this deviates from the conclusions of existing mainstream research - which mostly concludes that flexible labor has a negative impact on company performance, arguing that it reduces a company’s ability to innovate (Reljic et al., 2021), dilutes its corporate culture (Tim et al., 2019), and undermines a company’s long-term performance (Rubery et al., 2016). The essence of this contradiction lies in the reshaping of the nature of flexible labor by technological evolution: the breakthrough development of AI technology is prompting flexible labor groups to break through the stereotypical positioning of “low-end laborers” and to absorb a large number of high-end talents with digital skills. They are not only able to quickly respond to market fluctuations and project demands in a modular manner, but also achieve a level of resource allocation efficiency unattainable by traditional hierarchical organizations through intelligent tools such as algorithmic collaboration and remote cooperation. This intelligent upgrade of the talent structure enables the flexible workers to retain the flexibility advantages of the traditional outsourcing model while also possessing the output characteristics of knowledge-intensive work, potentially demonstrating greater adaptability and creativity than the formal employees. This transformation prompts us to re-examine the value proposition of flexible employment—it is no longer merely a tool for adjusting labor costs, but is emerging as a strategic choice for companies to build resilient organizational structures and capture the dividends of cutting-edge technologies.

The number of intelligent machine workers accounts for the lowest percentage, about 10% or less, but their contribution rate is the highest, reflecting their highest level of efficiency and output capacity, illustrating the great potential of intelligent and automation technologies in the automotive manufacturing industry. Intelligent machine workers represent advanced technological productivity, which means that they are capable of performing high-precision, high-intensity tasks with far greater efficiency than human employees (Li and Xu, 2020; Li et al., 2018). Specifically, intelligent machine workers are able to significantly reduce production cycle times through automated and intelligent workflows; and intelligent machine employees are capable of collecting and analyzing production data in real-time. This data reflects multi-dimensional information such as business processes, market dynamics, and user behavior, exerting a “multiplier effect” on the technological advancement of companies and thereby promoting a leap in productivity levels (Yang et al., 2022). With the advancement of technology and the continuous development of intelligent manufacturing, the proportion of intelligent machine workers in companies is expected to further increase. Companies should increase their investment in intelligent technologies to enhance production efficiency and output performance.

Based on the above conclusions, automobile manufacturing companies have optimized their human resource structure through blended human resource allocation to improve overall productivity and company performance. While formal employees ensure the stability and continuity of the company, flexible workers and intelligent machine workers bring higher production efficiency and responsiveness to the company. As flexible workers and intelligent machine workers are more efficient and productive than formal employees, the share of these two types of employees in companies is expected to increase further as technology advances and intelligent manufacturing continues to develop.

### 4.4 Further analysis

#### 4.4.1 Calculation of contribution rate of blended human resources based on the production function

As a classic economic analysis paradigm describing the relationship between inputs and outputs, the production function is an important tool for measuring the contribution rate of company human resources investment to output (Wu, 2012; Song and Guo, 2017). Based on the Cobb Douglas production function form, this paper constructs the expression of company blended human resource production function, analyzes the data of automobile manufacturing companies, and calculates the contribution rate of each type of production factor input to output in this industry. And the results are compared and analyzed with the calculation results based on BP-MIV above. The expression of blended human resource production function is constructed as follows:


(7) $$Q=A{L_{1}}^{\alpha_{1}}{L_{2}}^{\alpha_{2}}{L_{3}}^{\alpha_{3}}K^{\beta }$$

In Equation 7, Q is the output of the company; $L_{1}$ , $L_{2}$ and $L_{3}$ are the inputs of formal employees, flexible workers workers and intelligent machine workers, respectively, and K is the fixed capital inputs. The substitution indexes of each variable are consistent with the indexing features in Table 1. $\alpha_{1}$ , $\alpha_{2}$ , $\alpha_{3}$ and β are the output elasticities of each input factor. The output elasticity of a particular type of blended human resource refers to the additional increment of output that can be generated by a one-unit increase in that blended human resource input. Thus, the output elasticity of each type of blended human resource reflects the contribution rate of that type of blended human resource to the output of the company. The process of calculating output elasticities is shown below.

Write Equation 7 in logarithmic form:


(8) $$\ln Q=A+\alpha_{1}\ln L_{1}+\alpha_{2}\ln L_{2}+\alpha_{3}\ln L_{3}+\beta \ln K$$

In Equation 8, let lnQ = $Q\prime ,\ln L_{1}$ = $L_{1}\prime ,\ln L_{2}$ = $L_{2}\prime ,\ln L_{3}$ = $L_{3}\prime ,\ln K$ = K′ , and construct the regression equation:


(9) $$Q\prime =A+\alpha_{1}L_{1}\prime +\alpha_{2}L_{2}\prime +\alpha_{3}L_{3}\prime +\mathrm{\beta K}\prime +\in^{t}$$

$\in^{t}$ in Equation 9 is the error term. When analyzing the 2022 automotive manufacturing company data based on this regression equation, the following issues were identified: First, due to the strong correlation between the evaluation method for flexible worker numbers and formal employee counts, these two variables exhibit a significant proportional relationship, resulting in severe multicollinearity. Second, multicollinearity diagnostics revealed that the variance inflation factors (VIFs) for formal employee numbers and intelligent machine worker numbers were 17.073 and 13.940, respectively, both exceeding the threshold of 10, confirming the presence of serious multicollinearity. To address multicollinearity, this study adopted the ridge regression method following the research design of Zhou and Hu (2019). The results, as shown in Tables 3, 4, indicate that when the ridge penalty coefficient (*k*) was set to 0.24, the variation in standardized coefficients stabilized, and the regularized *R*^2^ reached 0.916, demonstrating excellent model fit. Accordingly, the coefficients for each variable in Table 4 were extracted and used as estimated contribution rates for blended human resource allocation, of which the contribution rates of formal employees, flexible workers are 20.1%, and intelligent machine workers is 14.7%. The final results are summarized in Table 5.

**Table 3:** Ridge trace results.

Number	Penalty (k)	Regularized R^2^	Sum of standardized coefficients	Apparent prediction error
1	0.000	0.932	1.000	0.068
2	0.020	0.932	0.469	0.068
3	0.040	0.931	0.251	0.069
4	0.060	0.930	0.254	0.070
5	0.080	0.928	0.236	0.072
6	0.100	0.927	0.237	0.073
7	0.120	0.925	0.189	0.075
8	0.140	0.924	0.195	0.076
9	0.160	0.922	0.183	0.078
10	0.180	0.921	0.171	0.079
11	0.200	0.919	0.166	0.081
12	0.220	0.918	0.162	0.082
13	0.240	0.916	0.158	0.084
14	0.260	0.915	0.155	0.085
15	0.280	0.913	0.151	0.087
16	0.300	0.912	0.148	0.088

Source(s): created by the authors.

**Table 4:** Ridge coefficients.

Number	Formal employee	Flexible workers	Intelligent machine workers	Fixed assets
1	−0.641	0.889	−0.001	0.682
2	−0.310	0.489	0.122	0.657
3	0.055	0.254	0.120	0.581
4	−0.008	0.292	0.148	0.563
5	0.023	0.274	0.156	0.542
6	−0.002	0.289	0.166	0.533
7	0.178	0.189	0.143	0.477
8	0.092	0.222	0.172	0.488
9	0.125	0.205	0.172	0.466
10	0.198	0.198	0.140	0.433
11	0.199	0.199	0.142	0.422
12	0.200	0.200	0.145	0.411
13	0.201	0.201	0.147	0.402
14	0.202	0.202	0.148	0.393
15	0.202	0.202	0.150	0.385
16	0.203	0.203	0.151	0.377

Source(s): created by the authors.

**Table 5:** Contribution rates of various production factors (calculated by production function model).

Label	1	2	3	4
Input variable	Formal employees	Flexible workers	Intelligent machine workers	Fixed assets
Contribution rate	20.1%	20.1%	14.7%	40.2%

Source(s): created by authors.

#### 4.4.2 Comparative analysis

Table 6 shows the comparative analysis based on the two methods (BP-MIV method and production function model) in the calculation of the contribution rate of companies in the automobile manufacturing industry. The results show that the production function model overestimates the contribution rate of fixed assets and underestimates the contribution rate of blended human resources (mainly intelligent machine workers). The reasons are (1) the production function fails to reflect the improvement of fixed asset productivity by intelligent technology. In the traditional production function model, fixed assets may be regarded as static capital inputs, but intelligent technologies, such as automated machines, AI systems, can enhance the productivity of existing fixed assets, allowing the same fixed asset inputs to produce greater outputs. Production function models that do not explicitly account for such efficiency gains may attribute the increase in output to the capital inputs themselves rather than to the efficiency improvements brought about by the technology, leading to an overestimation of the contribution of fixed assets. (2) The production function ignores the synergistic effects of blended human resources. Production function models decompose labor into different components, such as formal employees, flexible workers, and intelligent machine workers. However, the model may assume that these labor components act independently of each other, ignoring the synergistic effects between them. For example, intelligent machine workers may need to collaborate with formal employees to maximize efficiency, and this synergistic effect may not be captured in traditional models, leading to an underestimation of the contribution rate of intelligent machines and an overestimation of the contribution of fixed assets. (3) Rigidity of production function modeling assumptions. If the elasticity of substitution of capital and labor changes in the actual production process due to the introduction of intelligent technology, but the model still assumes fixed elasticity, it will lead to parameter estimation bias. For example, intelligent technology may make it easier for fixed assets to substitute for labor, but the model fails to reflect this change, thus overestimating the contribution of fixed assets.

**Table 6:** Comparison of the results of the two calculation methods.

Methods/indicators	BP-MIV method	Production function model	Extent of variation
Fixed assets contribution rate	26.19%	40.2%	+14.01%↑
Total blended human resource contribution rate	73.81%	54.7%	−19.11%↓
Formal employee contribution rate	19.55%	20.0%	+0.45%
Flexible Workforce contribution rate	20.26%	20.0%	−0.26%
Intelligent machine workers contribution rate	34.00%	14.7%	−19.3%↓

Source(s): created by authors.

## 5 Conclusion

A method based on BP-MIV for calculating the contribution rate of a company’s blended human resources is proposed. Based on this method, an empirical analysis was conducted on the blended human resource contribution rate of the automobile manufacturing industry, using data from companies in this sector from the year 2022 as the sample. The results show that the contribution rate of fixed assets to company performance in this industry is 26.19%, reflecting the importance of physical capital in the traditional manufacturing industry. However, what is more significant is that the contribution rate of blended human resources to company performance is as high as 73.81%, a finding highlighting the fact that blended human resources play a central role in modern corporate output. Within blended human resources, the contribution rate of different types of employees is different: 19.55% for formal employees, 20.26% for flexible workers, and 34.00% for intelligent machine workers. This outcome not only underscores the critical role of flexible and intelligent machine workers in elevating organizational performance, but also highlights the strategic imperative for companies to cultivate a more diversified and inclusive workforce. By embracing this model, organizations can better leverage complementary strengths across human and machine intelligence, enhance adaptive capacity in dynamic markets, and establish a sustainable competitive advantage in the intelligent era. In order to verify the rationality of the method, this study compares the calculation results of the production function-based blended human resource contribution rate calculation method with the BP-MIV-based blended human resource contribution rate calculation method of the company in further analysis sector. The results show that the BP-MIV-based calculation method is superior in capturing nonlinear relationships, such as the contribution of the synergies of blended human resources to the company performance, and to some degree makes up for the shortcomings of the production function-based calculation method.

Based on the above conclusions, the following suggestions are made: (1) pay attention to the allocation and optimization of blended human resources. Companies should actively explore and promote flexible employment modes, such as part-time work, remote work, and project cooperation, in order to attract more excellent talents and reduce labor costs. In addition, increase investment in intelligent technology, such as the introduction of automated production lines, intelligent robots, big data analysis, etc.; (2) optimize the management of formal employees. Companies can enhance the output level of formal employees by providing a good working environment, welfare benefits and career development opportunities, and strengthening training; (3) establish a scientific performance evaluation system. Companies should focus on exploring AI algorithms based on methods such as BP-MIV to establish a more scientific and comprehensive performance evaluation system that accurately evaluate the specific contribution of various types of employees to company performance. (4) Address Ethical Governance of AI Workforce Integration. To mitigate ethical challenges arising from AI’s deep integration into corporate HR systems, company should establish comprehensive governance measures including job displacement buffer mechanisms such as reskilling programs for AI-affected positions, algorithmic decision-monitoring frameworks featuring human oversight rights, and shared social responsibility models that proactively disclose AI’s employment impacts, thereby promoting sustainable human-AI collaboration in the workplace.

However, this study has the following limitations: (1) Conceptual level. Although the human-machine collaboration phenomenon was observed and neural networks were employed to capture the interaction effects among blended human resources, the intrinsic mechanisms were not revealed, nor were empirical tests conducted. Furthermore, the omission of key variables such as technological capital investment and relevant organizational factors may lead to biased contribution rate estimates, limiting the in-depth analysis of the collaborative mechanisms of blended human resources. (2) Methodological level. On one hand, core variables were indirectly estimated—the number of flexible workers was derived from secondary data and estimation methods, while the number of robots was allocated to individual company based on industry-wide data and historical employee figures. Although this approach has some theoretical foundation (Acemoglu and Restrepo, 2018; Li et al., 2021; Song and Hu, 2024; Wang J. et al., 2022; Wang and Dong, 2023), the lack of micro-level company evidence may introduce significant errors. On the other hand, the data were sourced from a single industry— the conclusions were drawn solely from samples in the capital-technology-intensive automotive manufacturing sector. Industries dominated by flexible labor (e.g., express delivery) or human-capital-intensive sectors (e.g., consulting) may exhibit different patterns, thus limiting the generalizability of the model.

Therefore, future research could be expanded in the following directions: (1) Theoretical framework extension. Incorporate variables such as company technological capital investment intensity (e.g., R&D expenditure ratio) and organizational contextual factors (e.g., management hierarchy complexity) to construct a more comprehensive calculation model for blended human resource contribution rates. Further deconstruct the heterogeneity of blended human resources (e.g., formal employees’ skill gradients, flexible worker contract types, autonomy levels of intelligent machines) to explore differentiated value creation patterns. Investigate the pathways through which blended human resources influence organizational performance, with particular attention to AI ethical conflicts (e.g., trade-offs between algorithm transparency requirements and efficiency objectives). (2) Empirical validation enhancement. First, test the generalizability of findings using cross-industry samples by selecting three representative sectors— express delivery (flexible labor-dominant), consulting (human capital-intensive), and electronics (technology-intensive)— and collecting company samples through stratified sampling to examine the domain adaptability of core conclusions. Second, complete company-level microdata collection by distributing structured questionnaires to HR and production sectors combined with semi-structured interviews with relevant employees to directly obtain blended workforce allocation data, thereby improving the accuracy of research outcomes. Third, compare the robustness of different machine learning models in contribution rate calculation by expanding beyond the current BP-MIV model to include heterogeneous algorithms like Random Forest (RF), Support Vector Machine (SVM), and Gradient Boosting Machine (GBM) as control experimental groups. Quantitative metrics will be used to assess significant differences in prediction accuracy and interpretability among models, providing objective validation for methodological selection.

## Data Availability

The original contributions presented in the study are included in the article/Supplementary material, further inquiries can be directed to the corresponding author.

## Footnotes

^1^The data on the total number of employees and the proportion of production personnel is sourced from the “Employee Structure Table of Publicly Listed Companies.” Data on fixed assets is derived from the “Fixed Assets, Accumulated Depreciation, and Impairment Provisions Table.” Revenue data is sourced from the “Revenue and Cost of Sales Table.”
