# Supplementary material for: AI-enabled workforce integration: blended human resource contribution rate in Chinese companies
Source: Front Artif Intell. 2025 Jul 9;8:1645172. doi: 10.3389/frai.2025.1645172 (PMC12285533; doi:10.3389/frai.2025.1645172)
Supplement: Supplementary file 1 [file Data_Sheet_1.pdf]

Appendix A Calculation process of the number of industrial robots in various companies within the automobile manufacturing industry in 2022

1. Model construction

Referring to the method of indirectly assessing the application of robots through the number of robot imports in existing literature (Li et al., 2021, Song and Hu, 2024, Acemoglu and Restrepo, 2020), based on the official data from the International Federation of Robotics(IFR), the industrial robot penetration of SAIC Motor is estimated, which in turn yields the amount of industrial robot in all years (Wang et al., 2022).

Step 1: Calculate the industrial robot penetration at the industry level.

$$Index_{st}^{CN} = \frac{MR_{st}^{CN}}{L_{s,t=2012}^{CN}} \tag{A1}$$

Where MR_{st}^{CN} is the stock of industrial robots in s industry in China's manufacturing industry in year t, and $L_{s,t=2012}^{CN}$ is the average total employment in s industry in China in 2012 (the base period).

Step 2: Calculate the industrial robot penetration at the company level:

$$Robot_{ist} = \frac{labor_{it=2012}}{alllabor_{s,t=2012}} \times Index_{st}^{CN} \tag{A2}$$

Where $labor_{it=2012}$ is the proportion of employees in the production sector to the total number of employees in manufacturing company i in 2012 (base period), and $alllabor_{s,t=2012}$ is the median proportion of production department employees to the total number of employees across all companies in industry s in manufacturing in 2012. The ratio of these two is used as a weight to scale the industry-level robot penetration down to the company level, excluding company-specific characteristics.

2. Data sources

2.1 IFR official data

The IFR has compiled statistics on the number of newly installed and operational industrial robots across various countries and industries. The number of industrial robots in the automotive manufacturing industry in China from 2012 to 2022 is shown in Appendix Table 1.

—— Appendix Table 1 is here ——

2.2 Employee data by industry

In 2011, the National Bureau of Statistics adjusted the 'Industrial Classification for National Economic Activities', dividing the transportation equipment manufacturing industry into two sectors: the automotive manufacturing industry and the railway, shipbuilding, aerospace, and other transportation equipment manufacturing industries. As a result, the transportation equipment manufacturing industry had not yet been subdivided in 2011. Moreover, data on employment in the various subdivisions of the manufacturing industry for 2012 had not yet been released. Since the average annual number of employees in the railroad, ship, aerospace and other transportation equipment manufacturing industry did not change significantly between 2013-2016, this paper adopts the 2013 data as the 2012 base period data(Wang et al., 2022), see Appendix Table 2.

—— Appendix Table 2 is here ——

2.3 Company-level data

The number of production employees and the total number of employees in automotive manufacturing companies are sourced from the personnel structure table in the CSMAR database.

3. Calculation results

Using equation (A1) and (A2), the industrial robot penetration $Robot_{ist}$ for automotive manufacturing companies in 2022 is calculated.

Reference

ACEMOGLU, D. & RESTREPO, P. 2020. Robots and jobs: Evidence from US labor markets. *Journal of political economy,* 128**,** 2188-2244.

LI, L., WANG, X. & BAO, Q. 2021. The Employment Effect of Robots: Mechanism and Evidence from China. *MANAGEMENT WORLD,* 37**,** 104-119.

SONG, J. & HU, X. 2024. How Robot Applications Improve the Green Development Quality of Chinese Manufacturing Enterprises — An Analysis Based on the Construction of Indices under the Dual Constraints of "Emission Reduction" and "Efficiency Enhancement". *MACRO ECONOMICS,* 12**,** 15-28.

WANG, J., WANG, Y. & SONG, J. 2022. Minimum Wage, Robot Application and Labor Income Share: Evidence from Listed Companies. *ECONOMIC RESEARCH JOURNAL,* 48, 106-120.

TABLES

Appendix Table 1. Number of industrial robots in the automotive manufacturing industry in China

Year	Industry Code	Industry Name	Number of new installations	Operational stock
2012	29	automobile manufacturing	11,429	32,247
2013			14,207	46,454
2014			21,106	67,560
2015			24,166	91,726
2016			25,679	117,405
2017			42,396	159,801
2018			39,351	198,753
2019			32,151	230,520
2020			30,000	260,520
2021			58,000	318,520
2022			73,000	391,520

Source(s): Compiled by the authors

Appendix Table 2. Number of employees by industry in 2013 (Unit: 10,000 People)

	Employment Figures
Total	14,025.8
Coal mining and washing	611.3
Oil and gas extraction	80.1
Ferrous Metal Mining and Processing	98.6
Nonferrous Metal Mining and Processing	76.2
Non-metallic Mining	124.9
Mining auxiliary activities	39.7
Other Mining	4.2
Agricultural Food Processing	584.1
Food Manufacturing	289.5
Alcohol, Beverage and Refined Tea Manufacturing	219.3
Tobacco Products	21.8
Textile Industry	663.7
Textile clothing and apparel industry	750.8
Leather, fur, feather and their products and footwear industry	441.9
Wood Processing and Wood, Bamboo, Rattan, Palm and Grass Products Industry	265.4
Furniture Manufacturing	199.1
Paper and paper products industry	219.8
Printing and recording media reproduction industry	195.1
Literary, Educational, Industrial, Sports and Recreational Goods Manufacturing Industry	371.6
Petroleum Processing, Coking and Nuclear Fuel Processing Industry	105.7
Chemical raw materials and chemical products manufacturing	655.3
Pharmaceutical manufacturing	242.7
Chemical fiber manufacturing	56.2
Rubber and plastic products industry	547.1
Non-metallic mineral products industry	987.8
Ferrous metal smelting and rolling processing industry	471.8

Non-ferrous metal smelting and rolling processing industry	243.5
Metal Products Industry	663.9
General Equipment Manufacturing	789.4
Specialty Equipment Manufacturing	580.2
Automobile Manufacturing	529.2
Railroad, ship, aerospace and other transportation equipment manufacturing industry	236.6
Electrical machinery and equipment manufacturing	844.2
Computer, communications and other electronic equipment manufacturing	1,028.3
Instrumentation Manufacturing	157.7
Other manufacturing industries	78.3
Comprehensive utilization of waste resources	29.9
Metal products, machinery and equipment repair industry	40.0
Electricity, heat production and supply industry	370.1
Gas Production and Supply	34.6
Water production and supply	76.3

Source(s): Official website of the China Statistics Bureau.
